# Epoetin β pegol (continuous erythropoietin receptor activator, CERA) is another choice for the treatment of anemia in myelodysplastic syndrome: a case report

**DOI:** 10.1186/s13256-017-1468-z

**Published:** 2017-10-19

**Authors:** Tatsuyoshi Ikenoue, Hiroshi Naito, Tetsuya Kitamura, Hideki Hattori

**Affiliations:** 1 Department of Healthcare Epidemiology, Kyoto University Graduate School of Medicine and Public Health, Yoshida Konoe-cho, Sakyo-ku, Kyoto, 606-8501 Japan; 2 Fujiidera Keijinkai Clinic, 1-2-3 Emisaka, Fujiidera, Osaka Japan; 3 Division of Hematology, Yao Municipal Hospital, 1-3-1 Ryugecho, Yao, Osaka Japan

**Keywords:** Myelodysplastic syndromes, Anemia, Erythropoietin, Renal dialysis

## Abstract

**Background:**

In most patients, anemia is present when myelodysplastic syndrome is diagnosed. Although darbepoetin α is the first-choice supportive therapy for low-risk myelodysplastic syndrome, half of all patients develop a loss of response to darbepoetin α within 12 months. However, few reports have described supportive therapy after the loss of response to darbepoetin α.

**Case presentation:**

We herein present a case involving a 65-year-old Japanese woman with low-risk myelodysplastic syndrome whose erythropoiesis-stimulating agent treatment was switched from darbepoetin α to epoetin β pegol (continuous erythropoietin receptor activator) to treat transfusion-dependent anemia. The frequent transfusions required to treat the anemia resulted in transfusion-associated circulatory overload. The transfusion-dependent anemia was initially treated with darbepoetin α, which negated the requirement for transfusion. However, after 12 months of darbepoetin α therapy, the hemoglobin concentration sharply declined. We switched her therapy from darbepoetin α to continuous erythropoietin receptor activator to avoid transfusion. After initiation of continuous erythropoietin receptor activator therapy, the hemoglobin concentration gradually increased and transfusion was not required. At the time of writing, no progression of the anemia had occurred.

**Conclusions:**

Although darbepoetin α is the first-choice supportive therapy for low-risk myelodysplastic syndrome, continuous erythropoietin receptor activator might be considered the second-choice therapy.

## Background

Myelodysplastic syndrome (MDS) is a malignant hematopoietic disease. Typical clinical features of MDS include ineffective hematopoiesis, which is caused by excessive premature apoptosis of hematopoietic precursors at disease onset [1]. The most frequently encountered type of cytopenia is anemia, which is present in approximately 70% of patients at the time of MDS diagnosis [2]. Anemia is also responsible for most symptoms of MDS. Anemia at the time of MDS diagnosis is an important prognostic factor according to the Revised International Prognostic Scoring System, which is the major risk classification system for MDS [3]. Transfusion is performed as supportive therapy to manage anemia, which leads to transfusion dependency in >80% of patients during the clinical course of MDS [4]. Transfusion dependency during the clinical course is an important independent prognostic factor. Transfusion-related iron overload is a critical condition predisposing to cardiac complications, increased fatigue, and decreased quality of life [3].

In clinical practice, erythropoiesis-stimulating agents (ESAs) are widely used to treat anemia in patients with low-risk MDS to reduce the risk of red blood cell transfusion [5]. The use of an ESA rather than transfusion improves the overall survival of patients with MDS [6–8], and patients with MDS who receive ESA therapy reportedly have longer survival times than those who do not receive ESAs [9, 10]. Darbepoetin α (DPO) is an ESA that reportedly improves the response rate of patients with MDS more than epoetin α and β [11–13]. In Japan, the use of DPO in patients with MDS has been covered by medical insurance since December 2014. Of the major responders to DPO, 10 to 50% experience a loss of response to DPO within 12 months [7, 14–17].

Few reports have described subsequent supportive therapy after the loss of response to DPO. Furthermore, to the best of our knowledge, no study has investigated the use of epoetin β pegol (continuous erythropoietin receptor activator, CERA) as supportive therapy in patients with MDS. In this case report, we describe the use of CERA as supportive therapy in a patient with MDS who experienced a loss of response to DPO through a switch from DPO to CERA.

## Case presentation

**Fig. 1:**
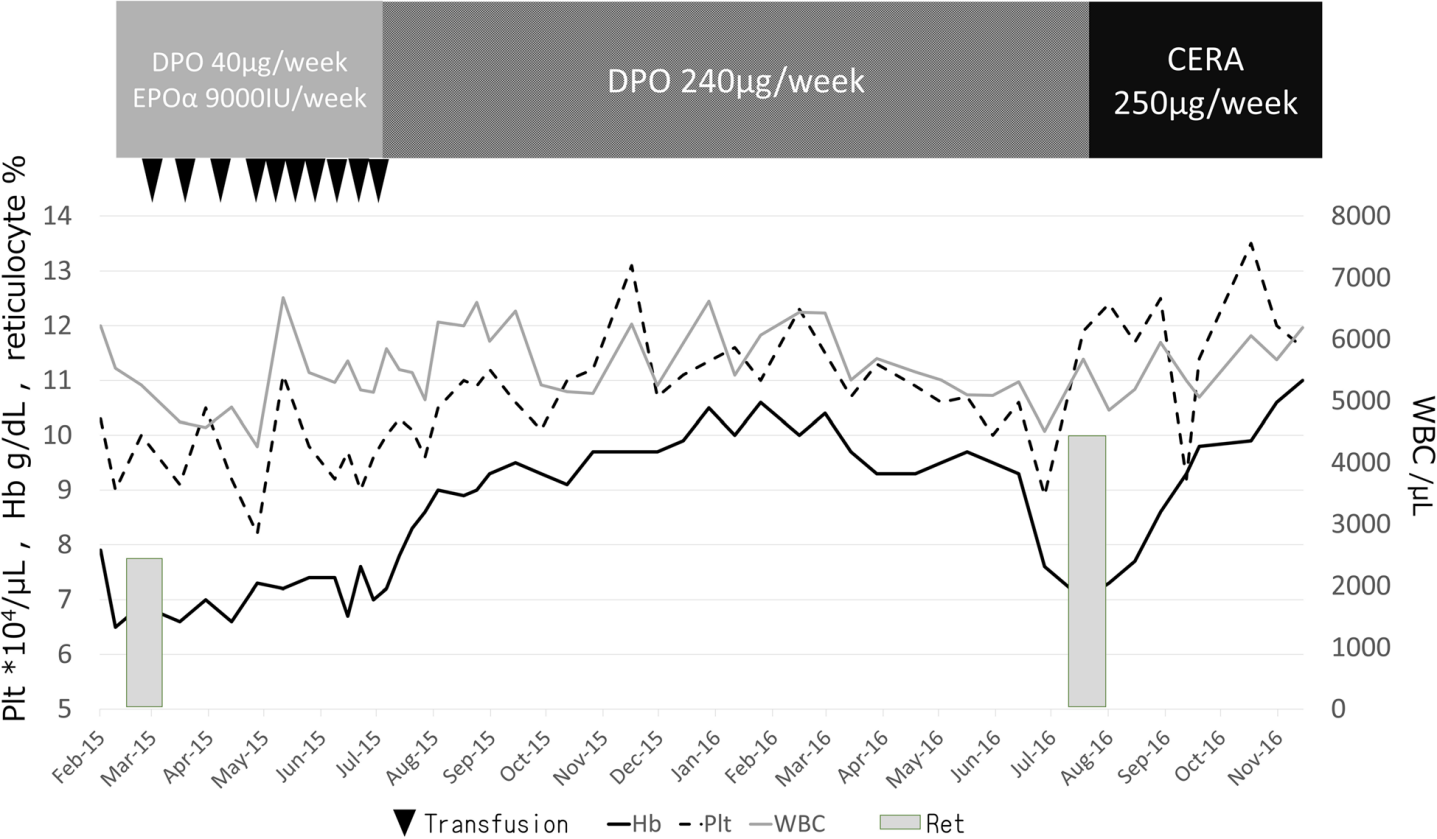
Clinical course of the patient. The hemoglobin concentration, platelet count, and white blood cell count are represented by *lines*. The percentage of reticulocytes is represented by *gray bars. CERA* continuous erythropoietin receptor activator (epoetin β pegol), *DPO* darbepoetin α, *EPOα* epoetin α

A 65-year-old Japanese woman diagnosed with MDS by bone marrow aspiration had been treated at a core hospital since 2008. She had refractory anemia as defined by the World Health Organization classification and was categorized in the low-risk group (Int-1) as defined by the International Prognostic Scoring System. A family history of anemia was absent. Our patient is a housewife and a nonsmoker. She is married with one daughter, and lives in an apartment in an urban area. She had a mastectomy of the left breast in 1986 with no recurrence, but no other relevant medical history. However, she developed diabetes in 1991 with resultant renal insufficiency. Her blood sugar control was suddenly aggravated at the time of MDS diagnosis. Hemodialysis had been initiated in January 2015, and she was transfusion-dependent with her hemoglobin concentrations being maintained at 6.5 g/dL by transfusion every 7 to 10 days before dialysis initiation. However, the frequent transfusions resulted in congestive heart failure, and she was hospitalized and diagnosed with transfusion-associated circulatory overload. She was transferred to our clinic for maintenance hemodialysis in February 2015. At this point, her serum erythropoietin level was low (127 mIU/mL), so she began treatment with epoetin α at 9000 IU/week plus DPO at 40 μg/week, which are the usual treatment dosages for renal anemia in our clinic. However, her anemia rapidly progressed while receiving ten transfusions during her dialysis sessions until June 2015; this treatment strategy was based on consultations with a hematologist. She continued to be transfusion-dependent, even while undergoing dialysis and treatment with a mid-range dose of ESAs. In July 2015, we increased the DPO to 240 μg/week to treat the anemia. After 2 weeks of DPO treatment, the anemia had resolved; she no longer needed transfusions and the hemoglobin concentration was maintained at >10 g/dL. However, from March 2016 she gradually developed resistance to the DPO treatment, and in July 2016 her hemoglobin concentration rapidly decreased to 6.8 g/dL. During March 2016, she had no obvious physical or neurological changes, but slight progression of conjunctival anemia. She was not feverous, and her average body temperature was 36.3 °C (97.34 F). Additionally, no changes were observed in her blood pressure or heart rate (average blood pressure, 144.0/56.5 mmHg; average heart rate, 60.6 beats per minute (bpm)). She underwent no changes in her medication and noticed no occupational changes associated with the decrease in hemoglobin. In July 2016, she developed a furuncle caused by a *Staphylococcus epidermidis* infection of her forearm, which was treated by gentamicin sulfate ointment within 5 days. The results of the laboratory findings are shown in Table 1. Differential diagnoses for this decrease in hemoglobin included gastrointestinal bleeding, pure red cell aplasia, infection, and iron deficiency; however, these were ruled out as causes of the decrease. We switched the ESA from DPO to CERA at 250 μg/week on 9 August 2016. After switching to CERA, the hemoglobin concentration gradually rose, and our patient no longer needed further transfusions. No progression of the anemia occurred for 1 year, and her hemoglobin concentration was stable at >10 g/dL (Fig. 1).

**Table 1:** Results of laboratory findings from March 2016

Before dialysis session		
*Complete blood count*		
White blood cell	5340	/μL
Lymphocytes	18.7	%
Basophils	0.4	%
Eosinophils	3.4	%
Neutrophils	72.6	%
Monocytes	4.9	%
Red blood cell	334	✕ 10^4^/μL
Hemoglobin	9.7	g/dL
Hematocrit	30.8	%
MCH	32.6	pg
MCHC	31.5	%
MCV	103	fL
Platelet	10.7	× 10^4^/μL
*Serology*		
C-reactive protein	0.13	mg/dL
Total bilirubin	0.6	mg/dL
Alanine transaminase	9	U/L
Aspartate transaminase	12	U/L
γ-glutamyltransferase	10	U/L
Alkaline phosphatase	244	U/L
Creatine kinase	41	U/L
Lactate dehydrogenase	156	U/L
Total protein	6.9	g/dL
Albumin	4	g/dL
Urea nitrogen	52.1	mg/dL
Creatinine	7.28	mg/dL
Uric acid	10.8	mg/dL
Sodium	141	mEq/L
Potassium	4.3	mEq/L
Chloride	104	mEq/L
Inorganic phosphorus	6	mg/dL
Calcium	8.1	mg/dL
Corrected calcium	8.1	mg/dL
Iron	48	μg/dL
Total iron binding capacity	181	μg/dL
Iron saturation	27	%
Ferritin	1736.1	ng/mL
Magnesium	2.4	mg/dL
Triglycerides	130	mg/dL
Low-density lipoprotein cholesterol	75	mg/dL
Blood sugar	271	mg/dL
Glycoalbumin	24.4	%
Parathyroid hormone-intact	358	pg/mL
β2-microglobulin	23.4	mg/L
After dialysis session		
*Serology*		
Albumin	4.5	g/dL
Urea nitrogen	13.9	mg/dL
Creatinine	2.22	mg/dL
Sodium	141	mEq/L
Potassium	2.8	mEq/L
Chloride	102	mEq/L
Inorganic phosphorus	2.1	mg/dL
Calcium	8.6	mg/dL
Atrial natriuretic peptide	34.6	pg/mL

## Discussion

In the present case, we determined that the cause of the decrease in hemoglobin was not related to progression of MDS, gastrointestinal bleeding, pure red cell aplasia, infection, and/or iron deficiency. Progression of MDS was ruled out by the stability of our patient’s white blood cell and platelet counts and lactate dehydrogenase concentration. Gastrointestinal bleeding was ruled out by gastrointestinal endoscopic examination and a fecal occult blood test. Pure red cell aplasia was ruled out by a high number of reticulocytes and the absence of anti-erythropoietin (EPO) antibody and anti-EPO receptor antibody using a commercial radioimmunoprecipitation assay. Although the sudden onset of DPO resistance is associated with infection, the infection was negligible in the present case (a minor shunt complication that was resolved with antibiotic ointment). Iron deficiency was also ruled out after testing. Hence, this decrease in hemoglobin was considered to be caused by the development of DPO resistance, which can occur during ESA use in patients with MDS.

EPO has been widely used as an anti-anemia agent in patients with end-stage renal disease since 1987 [18]. The introduction of EPO therapy markedly decreased the necessity of transfusion in patients with end-stage renal disease. DPO has a hyperglycosylated structure, giving it a longer elimination half-life and allowing for an extended dosing interval [19]. The most recently developed ESA, a third-generation drug known as epoetin β pegol or CERA, has a methoxy polyethylene glycol chain integrated via amide bonds between the N-terminal amino group of lysine using a succinimidyl butanoic acid linker [20]. CERA has the longest duration of action among all ESAs. Every ESA is widely used for patients undergoing dialysis, and the costs are included in a bundled dialysis treatment payment in Japan and the United States [21]. The use of ESAs among patients undergoing hemodialysis is summarized in Table 2 [22].

**Table 2:** Use of erythropoiesis-stimulating agents among patients undergoing hemodialysis

	Japan	United States	Europe
Available ESAs in HD therapy	Epoetin, darbepoetin, epoetin β pegol	Epoetin, darbepoetin, epoetin β pegol	Epoetin, darbepoetin, epoetin β pegol
Target range of hemoglobin	10–12 g/dL	10–12 g/dL	10–12 g/dL
Payment of ESAs among HD patients	Bundle	Bundle	Per dose
Mean (median) of ESAs dose in a week	5848 (5000)	13,834 (8655)	8216 (6249)

ESA dose conversions: subcutaneous epoetin × 1.15; darbepoetin (intravenous or subcutaneous) × 250 units/mg; and epoetin β pegol (intravenous or subcutaneous) × 208 units/mg

*ESA* erythropoiesis-stimulating agents, *HD* hemodialysis

Switching ESAs from EPO to DPO can resolve transfusion dependency in patients with MDS, as in the present case. In a comparison of the CERA dose before and after switching to DPO, the CERA-equivalent dose of EPO (250 μg of CERA = 52,000 IU of EPO) was lower than the DPO-equivalent dose of EPO (240 μg of DPO = 60,000 IU of EPO) [22]. With respect to the mechanism of action of ESAs, there are some differences between EPO and DPO. For example, although DPO exhibits the same mechanism of action as EPO [23], DPO has a threefold longer circulating half-life and is more potent *in vivo* than EPO [24]. This longer circulating half-life provides the benefit of DPO via preventing apoptosis and sustaining erythroid differentiation of erythroid precursors [25–27] as well as inducing globin gene expression and specifically promoting late erythroid differentiation in cooperation with GATA-1 [28]. Similar differences may also exist between DPO and CERA because CERA has a considerably longer half-life (139 h) than DPO (21 h) [29]. Because these differences do not depend on hemodialysis, switching ESAs from EPO to DPO can be expected to be effective in patients who are not undergoing hemodialysis.

Although switching to DPO from CERA has been reported in a case of pure red cell aplasia in a hemodialysis patient [30], switching to CERA from DPO has not been documented. However, the possibility of switching to CERA, which is associated with a very low rate of adverse events [31], can be considered among patients who develop a loss of response to DPO. This is a unique aspect of this case compared with previous reports in the literature. Although DPO is the first-choice supportive therapy in patients with MDS [32], we consider CERA to be the second-choice therapy before lenalidomide. Several studies have shown overall response rates ranging from 25 to 35% with an expected duration of response of 12 to 18 months [33–35]. The combination of lenalidomide and EPO significantly improves the erythroid response over lenalidomide alone in patients with lower-risk non-del5q MDS with ESA-resistant anemia [36]. In one study, however, grade 3/4 adverse events such as neutropenia (30%) and thrombocytopenia (25%) were common among patients using lenalidomide [37].

## Conclusions

In patients with low-risk MDS who have stopped responding to DPO for anemia, switching to CERA might help to avoid transfusion; however, the accumulation of more experience and knowledge of the use of CERA in patients with MDS is necessary. We hope that physicians who read this report will consider CERA as an alternative treatment option for anemia caused by MDS and that they will report case series involving CERA as the second option for treatment of MDS-induced anemia.

## Abbreviations

CERA: Continuous erythropoietin receptor activator
DPO: Darbepoetin α
EPO: Erythropoietin
ESAs: Erythropoiesis-stimulating agents
MDS: Myelodysplastic syndrome
